# Photoluminescence behavior of rare earth doped self-activated phosphors (*i.e.* niobate and vanadate) and their applications

**DOI:** 10.1039/d3ra00629h

**Published:** 2023-05-31

**Authors:** A. Dwivedi, A. Roy, S. B. Rai

**Affiliations:** a Sunbeam Women's College Varuna Varanasi-221002 India abhiphydwivedi731@gmail.com; b Department of Physics, Banaras Hindu University Varanasi-221005 India sbrai49@yahoo.co.in

## Abstract

In the present study, the photoluminescence behaviors of rare earth doped self-activated phosphors are discussed briefly. Different techniques were used to develop these phosphor samples. We prepared pure and rare earth doped phosphor samples to look for their various applications. The structural confirmations and surface morphologies were performed using X-ray diffraction (XRD) and scanning electron microscopy (SEM) measurements, respectively. The upconversion (UC) phenomenon was investigated in Tm^3+^/Yb^3+^ and Ho^3+^/Yb^3+^ co-doped niobate and vanadate based phosphors, which gave intense blue/NIR and green/red emissions with a 980 nm diode laser as an excitation source. Pure niobate and vanadate phosphor materials are self-activated hosts which give broad blue emission under UV excitation. Upon UV excitation, intense broad blue emission along with sharp emissions due to Tm^3+^ and Ho^3+^ ions are observed *via* energy transfer between niobate/vanadate and rare earth ions. These self-activated hosts show prominent downshifting (DS) behavior. Broad band quantum cutting (QC) was observed in these self-activated hosts, in which a blue emitting photon is converted into two NIR photons by co-doping Yb^3+^ ions in it. The multimodal (upconversion, downshifting and quantum cutting) behaviors of these phosphors make them very promising in various applications, such as spectral converters to enhance the efficiency of a c-Si solar cell, security ink and color tunable materials.

## Abstract 2

Rare earth doped self-activated phosphors show multimodal behavior applicable in color tunability, spectral converters and security ink.

## Graphical abstract

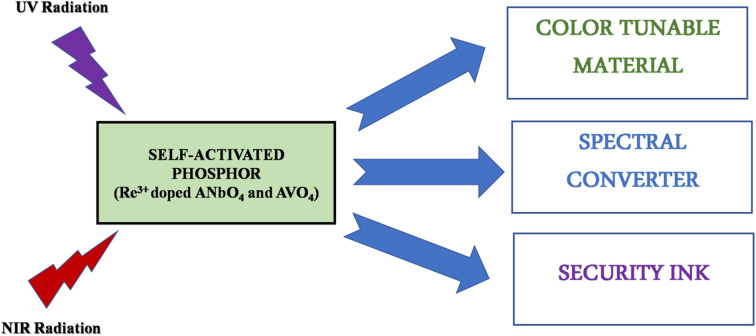

## 1. Introduction

Phosphors are materials that possess the phenomenon of luminescence. These materials have various applications in the fields of physics, chemistry, biology and material sciences. Self-activated hosts give their own intense emission in the visible region. Thus, these self-activated phosphors play a very crucial role for the excitation of different activators doped in them. Phosphors doped with rare earth ions have various applications, such as lasers, LEDs, sensors, display devices, bio-imaging, security ink, and color tunable materials.^1–12^ In this work, two types of hosts, ANbO_4_ and AVO_4_ (where A = Y, Gd and La), are investigated. These host materials are chemically, thermally, and mechanically stable and give intense broad blue emission on UV excitation. Most of the rare earth ions absorb radiation in the blue region and therefore their photoluminescence intensity on doping in these host materials is enhanced. This makes them useful for various applications. This blue emission is also helpful in white light generation as well as in achieving a color tunable radiation source. The downshifting (DS) behavior of the rare earth ions is enhanced significantly due to the self-activation behavior of the host.

ANbO_4_ (A = Gd, Y, La) with fergusonite structure was described by Blasse^13,14^ as early as 1976. The niobate group [(NbO_4_)^3−^] in a matrix acts as a fluorescence center and gives blue emission under UV excitation. YNbO_4_ and LaNbO_4_ show efficient blue emission, whereas GdNbO_4_ shows relatively weak blue emission on UV excitation. Since very little information is available about the luminescence properties of GdNbO_4_ based phosphors, it is interesting to study in detail this material and its properties. In GdNbO_4_, the niobium atom is attached tetrahedrally to the oxygen atom in a highly distorted site.^15–18^ AVO_4_ (A = Y and Gd) is a wide band gap host material since vanadate is a self-activated host which gives broad blue emission on UV excitation due to the presence of the (VO_4_)^3−^ group.^19–22^

Different rare earth ions (Tm^3+^, Ho^3+^, Yb^3+^) were used as activators doped into these self activated hosts. These activators have different spectral luminescence behavior in different regions. In this study, rare earth ion doped ANbO_4_ and AVO_4_ phosphors are discussed in terms of their multimodal properties and applications. For the multimodal behavior, we focused particularly on the upconversion (UC), downshifting (DS) and quantum cutting (QC) processes and looked for appropriate applications. For this purpose, Ho^3+^ and Tm^3+^ ions were used due to their ladder-like energy levels and intense optical properties.

## 2. Experimental methods

### 2.1. Materials used

All the starting materials used for the synthesis of the rare earth doped ANbO_4_, AVO_4_ and ATaO_4_ (where A = Y, Gd and La) phosphors were in oxide form. ABO_4_ phosphor materials can be synthesized by several techniques, such as the sol–gel method, solid state reaction method, combustion method, *etc.* Here, we used the solid state reaction method as it is easy.

### 2.2. Synthesis technique

All the phosphor samples were synthesized *via* the solid state reaction technique. Here, all the raw materials in oxide form were mixed homogeneously in an agate mortar for one hour using acetone (about 50 ml) as the mixing media. The raw mixtures were then calcined separately in an electric furnace at optimized higher temperatures (1400–1600 K) for 4–5 hours. The dry powders were then cooled to room temperature and finally ground into powder form using an agate mortar. These synthesized powder samples were used for the structural and optical characterizations.

## 3. Structural behavior

### 3.1. ANbO_4_ (A = Y, Gd and La)

**Fig. 1:**
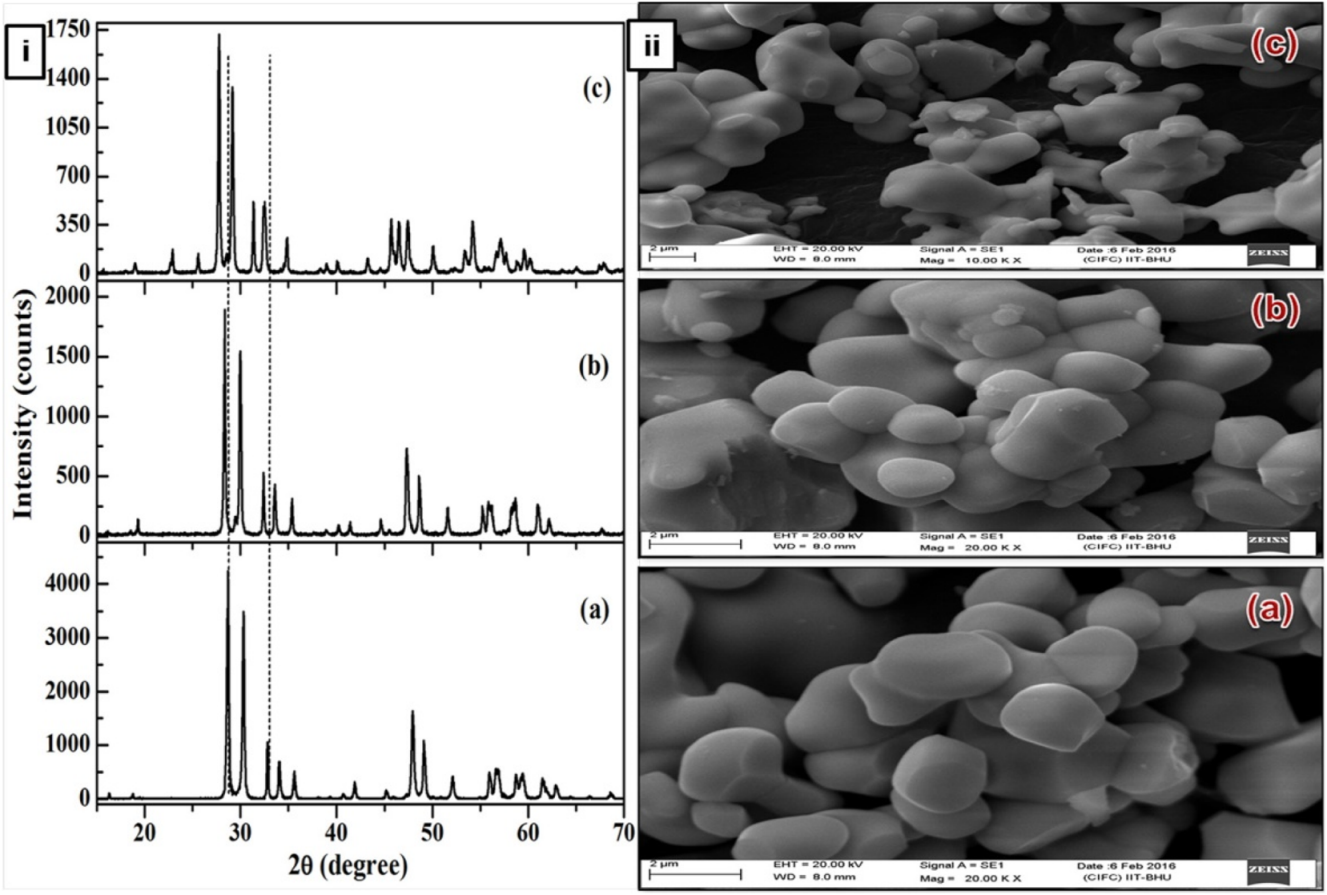
(i) X-ray diffraction patterns of (a) YNbO_4_, (b) GdNbO_4_ and (c) LaNbO_4_ phosphors. (ii) SEM images of (a) YNbO_4_, (b) GdNbO_4_ and (c) LaNbO_4_ phosphors. (Reproduced with permission from ref. 30 with copyright 2016, Institute of Physics).

ANbO_4_ (A = Y, Gd and La) compounds exhibit a fergusonite crystal structure and are thermally and chemically very stable. Lanthanide-activated YNbO_4_ crystallizes in a monoclinic phase with a space group of *C*2/*c* and lattice parameters of *a* = 7.6176(1) Å, *b* = 10.9454(2) Å, *c* = 5.2967(1) Å and *β* = 138.4308(8),^23,24^ as shown in Fig. 1(a). Lanthanide-activated GdNbO_4_ possesses an *I*2 space group in the monoclinic cell with refined lattice parameters of *a* = 5.3674(3) Å, *b* = 11.0772(6) Å, *c* = 5.1039(3) Å, and *β* = 94.554(4) (Fig. 1(b)).^25^ Similarly, lanthanide-activated LaNbO_4_ also crystallizes with monoclinic structure but with space group *I*2/*a* and lattice parameters *a* = 5.2297(1) Å, *b* = 10.8270(1) Å, *c* = 5.0416(1) Å, and *β* = 94.4029(6).^26^ In the monoclinic crystal structure, each A^3+^ ion coordinates with eight oxygen ions to form a dodecahedron and remains located at *C*_2_ symmetry sites. The Nb^5+^ ions remain coordinated with four oxygen ions to form a tetrahedral structure.

The SEM images show that the surface particles are well separated from each other and are of nearly spherical shape. Particles are similar in shape and size in both the YNbO_4_ and GdNbO_4_ phosphors. However, in the case of LaNbO_4_, the particles are agglomerated with each other and smaller in size compared to other two phosphors.

### 3.2. AVO_4_ (A = Y and Gd)

**Fig. 2:**
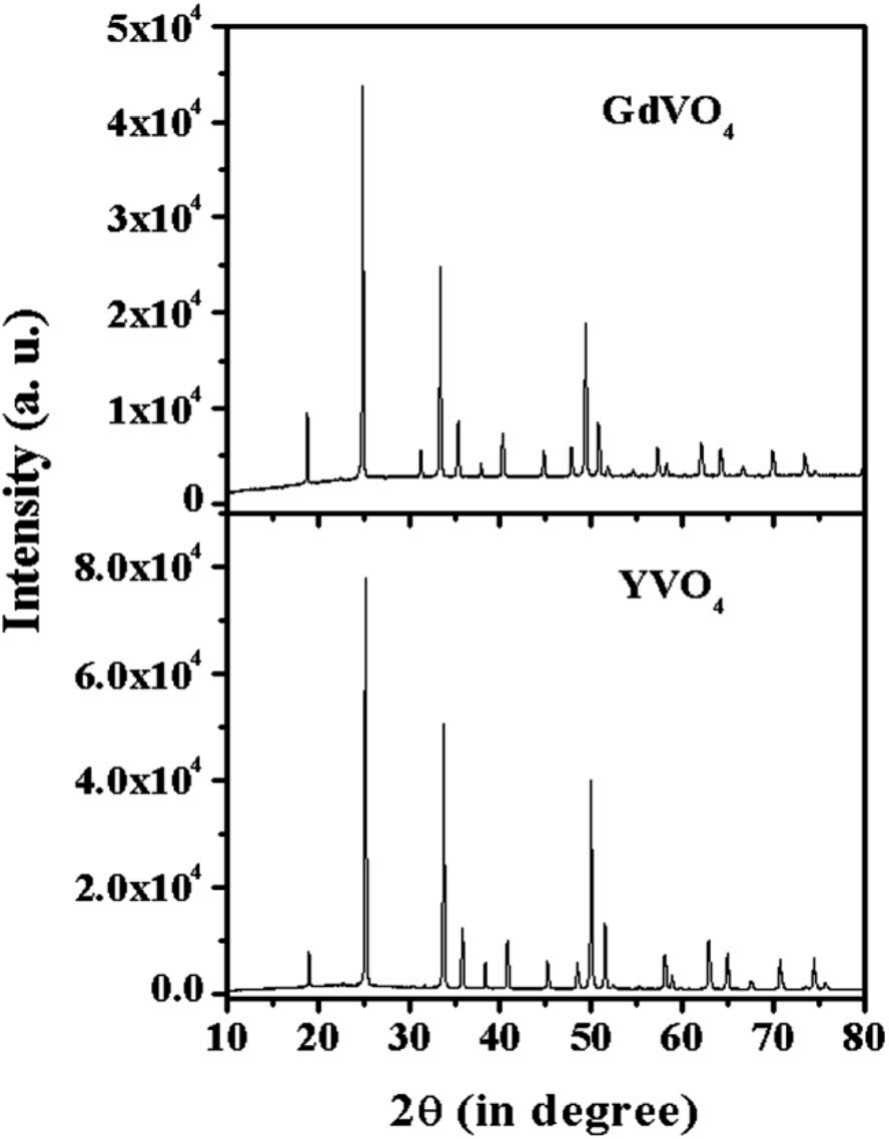
X-ray diffraction patterns of YVO_4_ and GdVO_4_ phosphors.

Fig. 2 shows the XRD spectra of the YVO_4_ and GdVO_4_ phosphors. The XRD pattern of YVO_4_ shows a tetragonal phase and matches well with JCPDS file no. 72-0274 with cell parameters of *a* = 7.100 Å, *c* = 6.270 Å and *α* = *β* = *γ* which confirm the pure phase formation of the prepared sample. The XRD peaks of the GdVO_4_ phosphor sample match well with JCPDS file no. 17-0260, confirming the tetragonal phase formation with cell parameters of *a* = 2.721 nm and *c* = 6.346 nm. The sharp and intense peaks of the XRD patterns show the pure crystalline nature of the synthesized samples.

## 4. Optical behavior

### 4.1. Upconversion (UC) behavior

#### 4.1.1. ANbO_4_:X^3+^,Yb^3+^ (where A = Y/Gd/La and X = Tm/Ho) phosphors

**Fig. 3:**
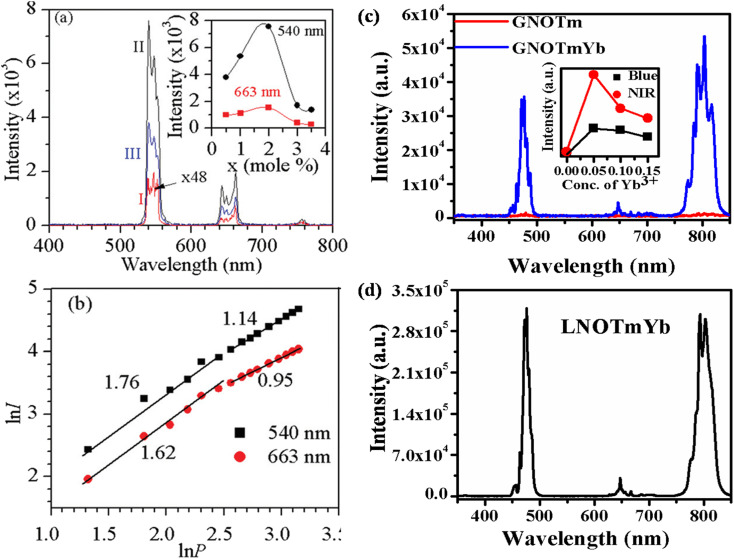
(a) UC emission spectra of GdNbO_4_:Ho^3+^,Yb^3+^ in the range 400–800 nm (I) without Yb^3+^ and (II) with Yb^3+^; the optimized green and red UC emission intensity with concentration of Ho^3+^ ion is shown in inset. (b) Ln *I versus* ln *P* plot of green and red emissions. (c) UC emission spectra of YNbO_4_:Tm^3+^ and YNbO_4_:Tm^3+^,Yb^3+^ phosphors in the range 350–850 nm; inset shows the optimized UC intensity with concentration of Yb^3+^ ion. (d) UC emission spectrum of LaNbO_4_:Tm^3+^,Yb^3+^ phosphor in the range 350–850 nm. ((a) and (b) are reproduced with permission from ref. 16 with copyright 2014, Royal Society of Chemistry).

Fig. 3 shows the UC emission behavior of the rare earth (Ho^3+^/Yb^3+^ and Tm^3+^/Yb^3+^) doped YNbO_4_, GdNbO_4_ and LaNbO_4_ phosphors in the visible region on excitation with a 980 nm diode laser.^25,28–30^ Intense green and moderately weak red emissions are observed in the GdNbO_4_:Ho^3+^,Yb^3+^ phosphor, as shown in Fig. 3(a), in which optimization was performed to get intense UC emission by varying the concentrations of Ho^3+^ and Yb^3+^ ions, shown in the inset of the figure. It was also observed that the incorporation of Yb^3+^ enhances the overall UC emission by several times (I and II spectra in the figure). Fig. 3(b) shows the power dependent study of UC emission, particularly in the green and red emissions, to understand the involvement of the number of photons by a log–log plot of green/red emissions with pump power. This shows that two photons are involved in the green and red emissions, as obtained from the slope. This slope decreases at higher pump powers due to the heating effect and the dominance of non-radiative relaxation. Similarly, Fig. 3(c) and (d) show the UC emission of the Tm^3+^/Yb^3+^ codoped YNbO_4_ and LaNbO_4_ phosphors in which strong blue and NIR emissions were observed at 476 (^1^G_4_ → ^3^H_6_) and 803 (^3^H_4_ → ^3^H_6_) nm, respectively. From Fig. 3(c), it can also be observed that the overall UC emission enhances significantly on the incorporation of Yb^3+^ ion, which suggests that Yb^3+^ also acts as a sensitizer ion.

#### 4.1.2. AVO_4_:X^3+^,Yb^3+^ (where A = Y/Gd and X: Ho/Tm) phosphors

**Fig. 4:**
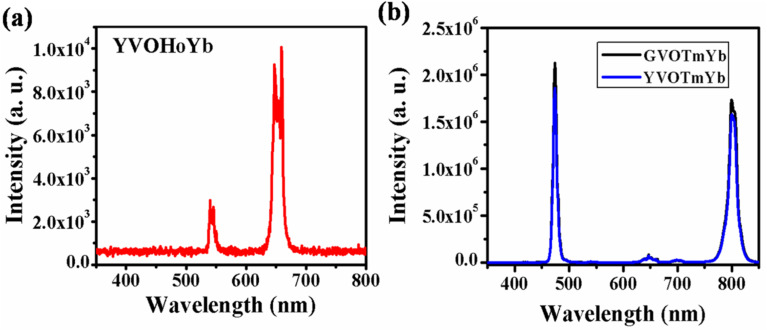
(a) UC emission spectrum of YVO_4_:Ho^3+^,Yb^3+^ phosphor in the range 350–800 nm. (b) UC emission spectra of Tm^3+^/Yb^3+^ co-doped GdVO_4_ and YVO_4_ phosphors in the range from 350–850 nm. ((b) is reproduced with permission from ref. 32 with copyright 2020, Elsevier).

Fig. 4(a) shows the UC emission spectrum of YVO_4_:Ho^3+^,Yb^3+^ phosphor in the range of 350–800 nm on excitation at 980 nm. In this case, weak green (540 nm) and intense red (663 nm) UC emissions are observed due to the ^5^F_4_/^5^S_2_ → ^5^I_8_ and ^5^F_5_ → ^5^I_8_ transitions, respectively.^31,32^ However, in the case of Ho^3+^/Yb^3+^ co-doped phosphors, green UC emission is dominant compared to red UC emission. Thus, this host is suitable for UC emission in the red region on excitation with a 980 nm diode laser.

Fig. 4(b) shows the UC emission spectra of the GdVO_4_:Tm^3+^,Yb^3+^ and YVO_4_:Tm^3+^,Yb^3+^ phosphors in the range of 350–850 nm on excitation with a 980 nm diode laser. Intense sharp blue (475 nm) and intense broad NIR (803 nm) UC emission lines are observed due to the ^1^G_4_ → ^3^H_6_ and ^3^H_4_ → ^3^H_6_ transitions, respectively. From the figure, it is observed that the UC emission of GdVO_4_:Tm^3+^,Yb^3+^ is more intense than that of the YVO_4_:Tm^3+^,Yb^3+^ phosphor due to their surface morphologies.^32^ In this host, the emission intensities of both the blue and NIR band are nearly equal, which is not observed in the niobate host due to the involvement of a different mechanism in the UC emission.

#### 4.1.3. Mechanism for the UC emission

**Fig. 5:**
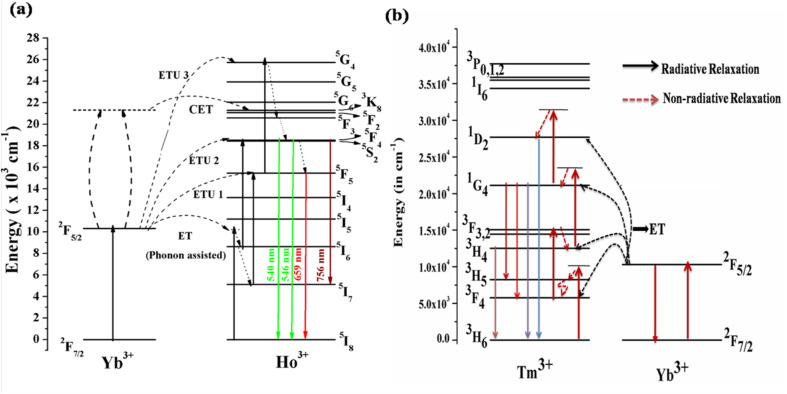
Energy level diagram of (a) Ho^3+^/Yb^3+^ions (reproduced with permission from ref. 19 with copyright 2019, American Chemical Society). (b) Tm^3+^/Yb^3+^ ions doped in ANbO_4_/AVO_4_ phosphor to reveal the UC emission mechanism (reproduced with permission from ref. 32 with copyright 2020, Elsevier).

Fig. 5(a) shows the energy level diagrams of the Ho^3+^/Yb^3+^ and Tm^3+^/Yb^3+^ ions to discover the mechanism behind the UC emission on excitation with a 980 nm diode laser. In these systems, the Yb^3+^ ion acts as a sensitizer and the Ho^3+^/Tm^3+^ ions as an activator. When the Ho^3+^/Yb^3+^ co-doped phosphor is irradiated by the 980 nm diode laser, the Yb^3+^ ion absorbs the incident radiation strongly due to it matching an energy level in Yb^3+^ and a higher cross-section of absorption.^33,34^ The de-excitation energy of the Yb^3+^ ion is transferred to the Ho^3+^ ion. Though the Ho^3+^ ion does not have a matching energy level, this is possible *via* phonon assistance. This energy transfer process is termed phonon-assisted energy transfer. Thus, the Ho^3+^ ion in the ground state (^5^I_8_) is promoted to ^5^I_6_*via* phonon-assisted energy transfer. The Ho^3+^ ions in the ^5^I_6_ level absorb the next photons and are promoted to the ^5^F_4_/^5^S_2_ level *via* the excited state absorption/energy transfer upconversion (ESA/ETU) process. The various transitions in the green and red regions are as follows.*hν* (980 nm) + Yb^3+^ (^2^F_7/2_) → Yb^3+^ (^2^F_5/2_)^2^F_5/2_ (Yb^3+^) + ^5^I_8_ (Ho^3+^) → ^2^F_7/2_ (Yb^3+^) + ^5^I_6_ (Ho^3+^)^2^F_5/2_ (Yb^3+^) + ^5^I_6_ (Ho^3+^) → ^2^F_7/2_ (Yb^3+^) + ^5^F_4_/^5^S_2_ (Ho^3+^)^5^F_4_/^5^S_2_ (Ho^3+^) → *hν* (green) + ^5^I_8_ (Ho^3+^)

This channel dominates in Ho^3+^/Yb^3+^ co-doped ANbO_4_ phosphors, giving intense green UC emission as compared to the red UC emission. Part of the energy in the ^5^F_4_/^5^S_2_ level relaxes non-radiatively to the close-lying ^5^F_5_ level and emits a red photon.

Another channel is also present in which UC emission from another excited level is possible. There are large numbers of quenching centers present in the system. One is non-radiative relaxation from ^5^I_6_ to ^5^I_7_*via* collisions. The ions in the ^5^I_7_ level absorb other photons and are promoted to the ^5^F_5_ level which is partly responsible for the red emission. The Ho^3+^ ion in the ^5^F_5_ level may be promoted *via* an ETU/ESA process. After absorbing a third photon, the Ho^3+^ ion is promoted to the ^5^G_4_ level. The relaxation from this high-lying level to the ^5^F_4_/^5^S_2_ and ^5^F_5_ levels gives emissions in the green and red regions. The mechanism can be represented as follows.^2^F_7/2_ (Yb^3+^) + *hν* → ^2^F_5/2_ (Yb^3+^)^2^F_5/2_ (Yb^3+^) + ^5^I_8_ (Ho^3+^) → ^2^F_7/2_ (Yb^3+^) + ^5^I_6_ (Ho^3+^)^5^I_6_ (Ho^3+^) → ^5^I_7_ (Ho^3+^) [*via* relaxation]^2^F_5/2_ (Yb^3+^) + ^5^I_7_ (Ho^3+^) → ^2^F_7/2_ (Yb^3+^) + ^5^F_5_ (Ho^3+^)^2^F_5/2_ (Yb^3+^) + ^5^F_5_ (Ho^3+^) → ^2^F_7/2_ (Yb^3+^) + ^5^G_4_ (Ho^3+^)

The Ho^3+^/Yb^3+^ co-doped AVO_4_ phosphor follows this mechanism to build up population in the higher levels. Therefore, the green emission is very weak compared to the red emission in the AVO_4_:Ho^3+^,Yb^3+^ phosphor.

Another channel for UC emission is cooperative energy transfer (CET) between two Yb^3+^ ions and one Ho^3+^ ion. In this process, two Yb^3+^ ions interact such that their deexcitation energy is transferred to the Ho^3+^ ion to simultaneously excite it to the ^5^F_4_/^5^S_2_ level. The relaxation from this level to lower levels gives transitions in various regions.2 ^2^F_5/2_ (Yb^3+^) + ^5^I_8_ (Ho^3+^) → 2 ^2^F_7/2_ (Yb^3+^) + ^5^F_2_ (Ho^3+^)

This channel is not as effective as the other two channels.

The Tm^3+^ ion doped alone in the phosphors shows very weak UC emission at only a high pump power density of the 980 nm diode laser through the ESA process. The efficiency of the UC emission can be enhanced by several times through adding Yb^3+^ ion as a sensitizer. As mentioned in the earlier section, the absorption cross-section of Yb^3+^ ion for 980 nm is very large compared to those of other rare earth ions. Yb^3+^ ion efficiently absorbs NIR photons (980 nm radiation) in its ground state (^2^F_7/2_) and is promoted to the excited state (^2^F_5/2_), as shown in Fig. 5(b). The de-excitation energy of the excited Yb^3+^ ion is transferred to the Tm^3+^ ion by two different possible paths. In the first process, the Yb^3+^ ion transfers its excitation energy non-resonantly (phonon assisted ET) to the Tm^3+^ ion, which is in its ground state (^3^H_6_), and excites it to the higher state (^3^H_5_). Since the lifetime of the ^3^H_5_ state is very short, the excited Tm^3+^ ions relax non-radiatively to the lower lying metastable ^3^F_4_ state where they reabsorb a second 980 nm photon (ESA) and are promoted to the ^3^F_2_ state. From there, the ions relax to the ^3^H_4_ state *via* the intermediate state ^3^F_3_. Finally, the Tm^3+^ ion in the ^3^H_4_ state absorbs a third 980 nm photon and is promoted to the ^1^G_4_ state. The Tm^3+^ ion in the ^1^G_4_ level gives different transitions to low lying states, emitting in different wavelength regions. This can be represented by the multistep ET from Yb^3+^ to Tm^3+^ as follows.*hν* (980 nm) + Yb^3+^ (^2^F_7/2_) → Yb^3+^ (^2^F_5/2_)^2^F_5/2_ (Yb^3+^) + ^3^H_6_ (Tm^3+^) → ^3^H_5_ (Tm^3+^) + ^2^F_7/2_ (Yb^3+^)^2^F_5/2_ (Yb^3+^) + ^3^F_4_ (Tm^3+^) → ^3^F_2_ (Tm^3+^) + ^2^F_7/2_ (Yb^3+^)^2^F_5/2_ (Yb^3+^) + ^3^F_3_ (Tm^3+^) → ^1^G_4_ (Tm^3+^) + ^2^F_7/2_ (Yb^3+^)

This process is called sequential sensitization. In the second mechanism, the sensitization process is known as cooperative sensitization, as discussed earlier. In this process, two excited Yb^3+^ ions in the excited state ^2^F_5/2_ after the absorption of a 980 nm photon interact with each other through dipole–dipole interaction, which is most effective. Due to this interaction, two emission photons from the excited Yb^3+^ are coupled to each other and this energy directly excites the Tm^3+^ ion to the excited level ^1^G_4_. From there, further emissions are possible due to different transitions to lower lying levels, *i.e.* the cooperative sensitization process.2 ^2^F_5/2_ (Yb^3+^) + ^3^H_6_ (Tm^3+^) → ^1^G_4_ (Tm^3+^) + 2 ^2^F_7/2_ (Yb^3+^)

### 4.2. Downshifting behavior

#### 4.2.1. ANbO_4_:Tm^3+^,Yb^3+^ (where A = Y, Gd and La)

**Fig. 6:**
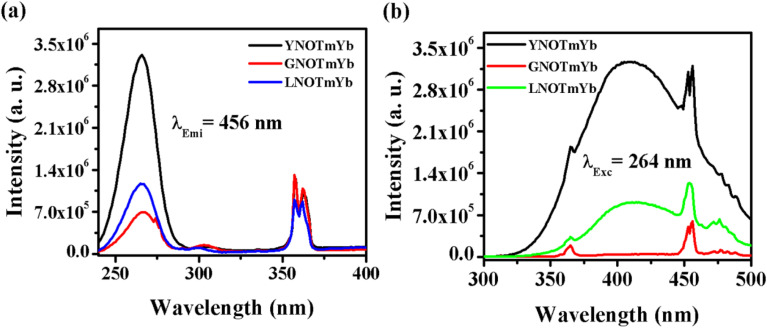
(a) Photoluminescence excitation and (b) photoluminescence emission spectra of ANbO_4_:Tm^3+^,Yb^3+^ (where A = Y, Gd and La) phosphors.

Fig. 6 shows the photoluminescence excitation (PLEx) and photoluminescence emission (PLEm) spectra of the ANbO_4_:Tm^3+^,Yb^3+^ (where A = Y, Gd and La) phosphors. We already discussed the self-activation behavior of niobate-based phosphors in which broad blue emission was observed on UV excitation. The PLEx spectra were recorded by monitoring emission at 456 nm due to the ^1^D_2_ → ^3^F_4_ transition in ANbO_4_:Tm^3+^,Yb^3+^ (ANOTmYb, where A = Y, Gd and La) phosphors in the range of 230–400 nm, as shown in Fig. 6(a). In the spectra, a broad band is observed at 264 nm due to the (NbO_4_)^3−^ group along with a sharp band at 362 nm due to the ^3^H_6_ → ^1^D_2_ transition of the Tm^3+^ ion. From the excitation spectra, it was confirmed that there is energy transfer from the (NbO_4_)^3−^ group to the Tm^3+^ ion. The excitation band at 264 nm is more prominent in YNOTmYb than in the others, while the 362 nm peak is more prominent in GVOTmYb. Fig. 6(b) shows the PLEm spectra of the ANbO_4_:Tm^3+^,Yb^3+^ (ANOTmYb, where A = Y, Gd and La) phosphors on excitation at 264 nm in which a broad blue emission is observed at 415 nm due to the (NbO_4_)^3−^ group along with a sharp emission at 456 nm due to the ^1^D_2_ → ^3^F_4_ transition of the Tm^3+^ ion. The emission is more intense in YNOTmYb due to the strong excitation band of the (NbO_4_)^3−^ group which is responsible for the strong energy transfer between the host and the Tm^3+^ ion.

#### 4.2.2. YVO_4_:Ho^3+^ phpsphor

**Fig. 7:**
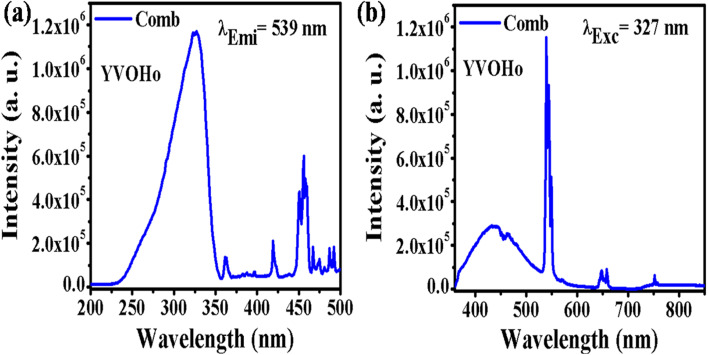
(a) Photoluminescence excitation and (b) photoluminescence emission spectra of YVO_4_:Ho^3+^ phosphor.

Fig. 7 shows the PLEx and PLEm spectra of the YVO_4_:Ho^3+^ phosphor. In this phosphor, a broad excitation band is observed at 327 nm due to the (VO_4_)^3−^ group, along with a sharp f–f transition due to the Ho^3+^ ion (Fig. 7(a)). This excitation spectrum was recorded by monitoring emission at 539 nm due to the ^5^F_4_/^5^S_2_ → ^5^I_8_ transition of the Ho^3+^ ion. The photoluminescence emission spectrum was monitored in the range of 350–850 nm on excitation at 327 nm in the YVO_4_:Ho^3+^ phosphor, as shown in Fig. 7(b). A strong emission band is observed at 440 nm due to the (VO_4_)^3−^ group along with sharp emissions at 539 and 659 nm due to the ^5^F_4_/^5^S_2_ → ^5^I_8_ and ^5^F_5_ → ^5^I_8_ transitions of the Ho^3+^ ion. There is a strong energy transfer from the (VO_4_)^3−^ group to the Ho^3+^ ion. Therefore, YVO_4_ is a self-activated host which gives strong broad blue emission on UV excitation. This broad blue emission along with the green and red emissions gives white light or even tunable light.

#### 4.2.3. Mechanism of downshifting emission

**Fig. 8:**
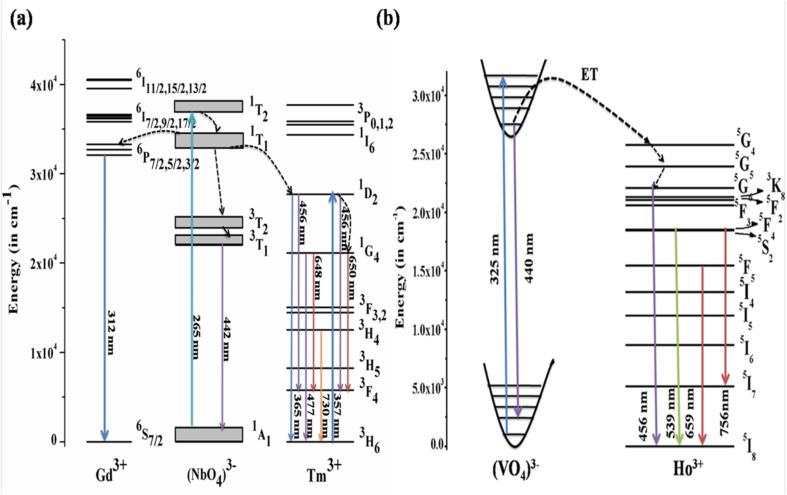
Energy level diagrams of (a) GdNbO_4_:Tm^3+^ (reproduced with permission from ref. 25 with copyright 2015, Institute of Physics) and (b) YVO_4_:Ho^3+^ phosphors (reproduced with permission from ref. 19 with copyright 2019, American Chemical Society).

The mechanism behind the downshifting emission in GdNbO_4_ is explained by the possible energy level diagram shown in Fig. 8(a). With *λ*_ex_ = 265 nm, the CTB of the NbO_4_ group is directly excited. This CTB excitation gives two results: first, radiative transition at 442 nm due to ^3^T_1_ → ^1^A_1_ of the NbO_4_ group and second, some excitation energy transfer to the Tm^3+^ ion and the Gd^3+^ ion. Thus, the Tm^3+^ and Gd^3+^ ions are excited due to ET from the NbO_4_ group and the characteristic emission peaks from the Tm^3+^ ion are observed. Due to transfer of part of the energy from the (NbO_4_)^3−^ group to the Gd^3+^ ion, the emission intensity of the (NbO_4_)^3−^ group is not more prominent compared to other two.

Similarly, the mechanism behind this DS behavior in YVO_4_:Ho^3+^ is well explained by the energy level diagram shown in Fig. 8(b). The (VO_4_)^3−^ group is excited at 327 nm. The emission from the (VO_4_)^3−^ group is observed in a broad blue region at 440 nm. There is resonant energy transfer from the (VO_4_)^3−^ group to the ^5^G_4_ level of the Ho^3+^ ion. The Ho^3+^ ions relax from that level to lower levels and give various emissions in different regions.

### 4.3. Quantum cutting (QC) behavior

In general, QC can be understood as a cascade emission of photons from a single rare earth ion or *via* ET through different sets of rare earth ions. NIR QC has been observed in various systems co-doped with Ln^3+^–Yb^3+^ (Ln = Tm, Pr, Er, Ho, Nd, Tb and Dy)^35–41^ and also in systems with single rare earth doping (*e.g.* Ho^3+^, Tm^3+^ or Er^3+^).^42–44^ In QC, there are two types of energy transfer (ET): (1) first order ET and (2) second order ET (cooperative sensitization process). In the first case, there is spectral overlap between the donor emission and the acceptor absorption. If there is no spectral overlap between the donor emission and the acceptor absorption, then second order ET becomes the dominant process, also called cooperative sensitization. In the second case, the sum of the energy of the absorption (excitation) of two acceptors must equal the energy of the donor emissions for the resonant condition.^6^

**Fig. 9:**
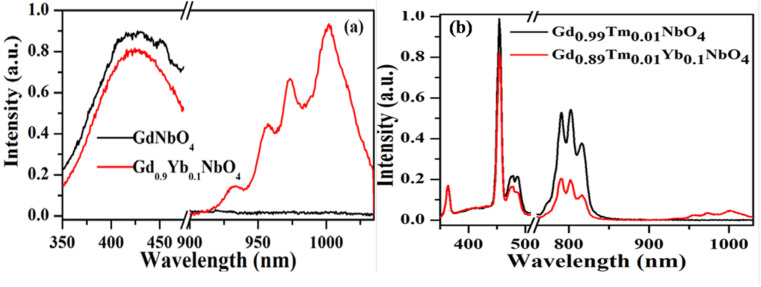
Photoluminescence emission spectra of (a) GdNbO_4_ and Gd_0.9_Yb_0.1_NbO_4_ and (b) Gd_0.99_Tm_0.01_NbO_4_ and Gd_0.89_Tm_0.01_Yb_0.1_NbO_4_ phosphors on excitation with 266 nm. (Reproduced with permission from ref. 25 with copyright 2015, Institute of Physics).

**Fig. 10:**
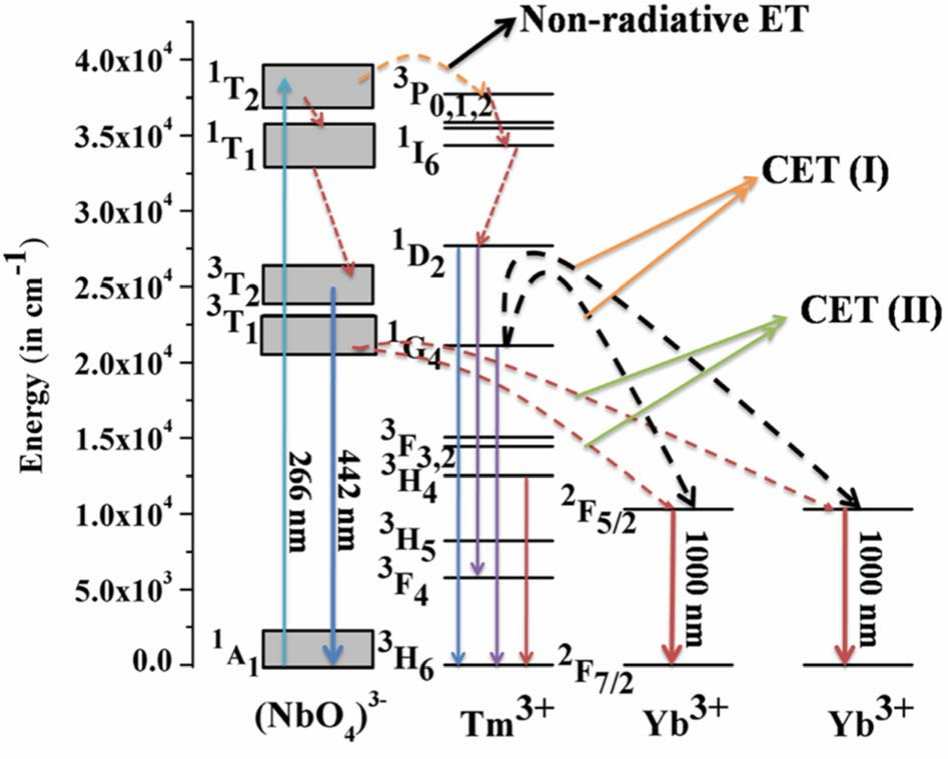
Energy level diagram to explain the mechanism of quantum cutting (QC) behavior. (Reproduced with permission from ref. 25 with copyright 2015, Institute of Physics).

Host matrices play a crucial role in the sensitization process. GdNbO_4_ is known as a self activated compound which has a charge transfer band (CTB) in the UV region and gives blue emission upon UV excitation.^25,30^ In GdNbO_4_, the niobate group [(NbO_4_)^3−^] acts as a fluorescence center in the matrix and gives blue emission under UV excitation. In the previous section, it was observed that there is a broad CTB at 266 nm and also a broad blue emission at 442 nm due to the (NbO_4_)^3−^ group since the resonance condition of CET from (NbO_4_)^3−^ to Yb^3+^ ion is well satisfied. Therefore, CET takes place and NIR emission (≈900–1040 nm) due to the Yb^3+^ ion was observed (Fig. 9(a)). This phenomenon of NIR QC by CET from the host to the acceptor ion has not been much explored. The possible mechanism of NIR QC can be explained in terms of the possible energy level diagram in Fig. 10. Upon 266 nm Nd:YAG laser excitation, the ^1^T_2_ level of the (NbO_4_)^3−^ group is populated. This population relaxes non-radiatively to the ^3^T_1_ level from which the blue emission (at 442 nm) is obtained. The emission from ^3^T_1_ to ^1^A_1_ of the (NbO_4_)^3−^ group (≈442 nm) excites two Yb^3+^ ions in the excited level ^2^F_5/2_. Then, the further transition from ^2^F_5/2_ to ^2^F_7/2_ gives NIR emission (≈900–1040 nm), as shown in Fig. 9(a).

Fig. 9(b) shows the emission spectra for the Gd_0.9_Tm_0.01_NbO_4_ and Gd_0.8_Tm_0.01_Yb_0.1_NbO_4_ phosphors using *λ*_ex_ = 266 nm. It can be observed that both spectra are similar, except the emission in the range of 900–1040 nm in the case of the Gd_0.8_Tm_0.01_Yb_0.1_NbO_4_ phosphor. The possible mechanism for the observed NIR emission can be explained as follows: with 266 nm excitation, the Tm^3+^ ion is promoted to the high lying level ^3^P_2_ as shown in Fig. 10. From there, the low lying level ^1^D_2_ is populated *via* non-radiative relaxation, then further to lower level ^1^G_4_. The lifetime of this level is relatively high and it behaves as a metastable state. From this level, blue emission is observed at 477 nm. There may be two possibilities of QC emission in Yb^3+^ ions from the ^1^G_4_ level: the first is cooperative energy transfer from the ^1^G_4_ level of the Tm^3+^ ion to two Yb^3+^ ions (since the energy of the ^1^G_4_ level is just double of the energy level of two Yb^3+^ ions) and the second is a cross relaxation process between ^1^G_4_ → ^3^H_5_ transition (Tm^3+^ ion) and ^2^F_5/2_ → ^2^F_7/2_ transition (Yb^3+^ ion).

## 5. Applications

### 5.1. As a spectral converter

**Fig. 11:**
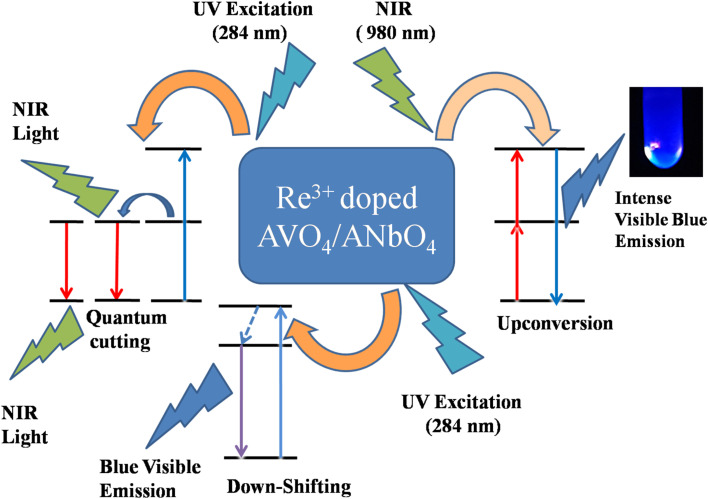
Schematic diagram to explain the spectral conversion process.

The multimodal (UC, DS and QC) behavior of Tm^3+^/Yb^3+^ or Ho^3+^/Yb^3+^ co-doped ANbO_4_/AVO_4_ phosphors suggests that such materials can be used as a spectral converter. In this, UV/visible light is converted to visible/NIR light *via* a DS process and NIR light is converted to visible light *via* a UC process. Along with these two processes, one very peculiar phenomenon is also observed in which one high energy photon (in the UV region) is converted to two or more low energy photons (in the visible or NIR region) *via* a QC process. These types of materials are very useful for the efficiency enhancement of c-Si solar cells due to the spectral conversion behavior.^27^Fig. 11 shows a schematic diagram to explain these spectral conversion processes.

**Fig. 12:**
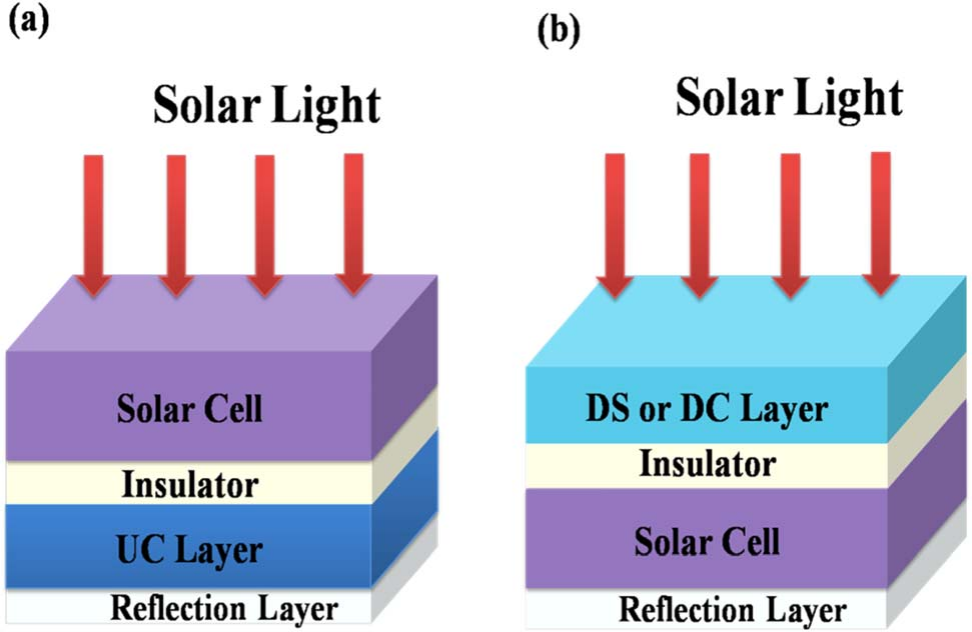
Schematic diagram of (a) a UC layer and (b) a DS or DC layer of phosphor material on a solar cell. (Reproduced with permission from ref. 25 with copyright 2015, Institute of Physics).

Energy losses due to thermalization of high energy photons higher than the band gap and to transmission of low energy photons less than the band gap can be minimized by downconverting (QC) high energy photons to low energy photons and by upconverting low energy photons from the solar spectrum to high energy photons, respectively. Trupke *et al.*^45,46^ discussed the efficiency of a solar cell equipped with a downconverting layer located on the front surface of a single junction solar cell and an upconverting layer on the rear side as shown in Fig. 12. A perfect reflector is located at the rear of the upconverter.

### 5.2. Security ink application

**Fig. 13:**
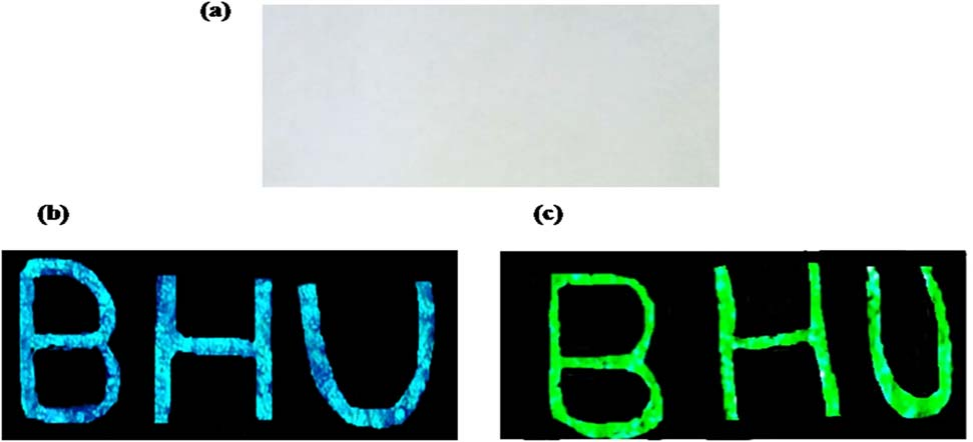
The paper with BHU written on it (a) in normal light illumination, (b) showing blue emission on excitation with 365 nm radiation and (c) showing green emission on excitation with 980 nm radiation. (reproduced with permission from ref. 5 with copyright 2020, Elsevier).

Rare earth doped self activated phosphors have potential application as security ink. As seen above, phosphor materials show very intense emission upon UV and NIR excitation. A security coding using this material can be coated on any document; it is not visible in normal light, but the coded security surface becomes visible when this document is exposed to UV or NIR radiation. The security ink applications of phosphor materials have been used for anti-counterfeiting applications. This technique makes items easier to authenticate from fake brands and documents. The optical emission properties of the phosphor are invariant when it is dispersed in acetone or ethyl alcohol. For the development of this type of security ink, we dispersed an appropriate amount of phosphor within a particular liquid medium. The main challenges in this field are high stability, good dispersion of the luminescent nanophosphor, the sticky nature of the ink medium on surfaces and the low cost and availability of the ink medium (Fig. 13).

### 5.3. Color tunability with variation of parameters (concentration, external power and external temperature)

**Fig. 14:**
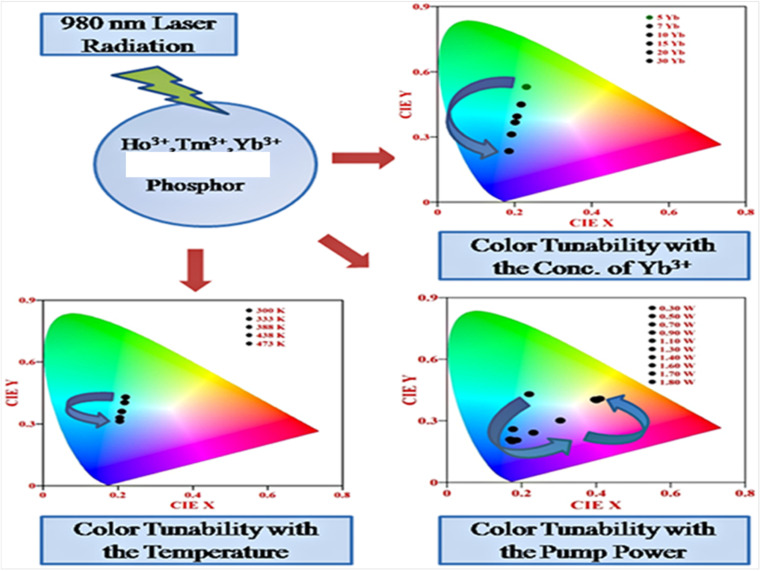
Color tunability of Ho^3+^/Tm^3+^/Yb^3+^ co-doped phosphors in the UC emission process when varying the concentration, pump power and external temperature.

Rare earth doped self activated phosphors show color tunable behaviors when varying the concentration of activators/sensitizers, pump power, and external temperature. These color tunable materials are currently used in various applications such as anti-counterfeiting, sensing, security, *etc.* In Fig. 14, the color tunability is shown in the UC emission process in Ho^3+^/Tm^3+^/Yb^3+^ doped in these hosts by plotting the CIE diagram in which the UC emission intensity can be tuned from light green to light blue by varying the concentration of Yb^3+^ ion. The color tunability is observed when varying the pump power of the diode laser in which the laser induced optical heating effect becomes dominant with the pump power and causes different relaxation processes. Due to these relaxation processes, there is color tunability with pump power. This color tunability was further investigated by varying the external temperature around the activator ion.

## 6. Conclusions

Self activated hosts play a very crucial role in the significant enhancement and variation of optical properties of the rare earth ions doped in these host materials. The ANbO_4_ (where A = Y, Gd and La) phosphor shows self activation behavior in which broad blue emission is observed from the host on UV excitation. Similarly, the AVO_4_ (where A = Y and Gd) phosphor also shows self activation behavior when monitoring the photoluminescence excitation and emission spectra. Tm^3+^/Yb^3+^ and Ho^3+^/Yb^3+^ co-doped niobate and vanadate phosphors show intense blue, NIR, green and red emissions *via* a UC emission process. DS behavior shows the self activation of ANbO_4_ and AVO_4_ phosphors and also shows the energy transfer process between the host and the activator ions (*i.e.*, Tm^3+^ and Ho^3+^ ions). The QC process is also observed in these self-activated phosphors and is a new and interesting phenomenon. Broad band QC is observed in ANbO_4_/AVO_4_ phosphors due to the self-activation behavior of the host. The multimodal behavior in such materials makes them useful for spectral conversion processes which have been frequently used for the enhancement of the efficiency of c-Si solar cells. The color tunable behaviors of these materials make them very useful in various display devices. Currently, luminescence tags are most the popular security elements for authenticating various items.

## Conflicts of interest

The authors declare that they have no known competing financial interests or personal relationships that could have appeared to influence the work reported in this manuscript.

## Supplementary Material
